# Understanding and Improving ^18^F-Fluciclovine PET/CT Reports: A Guide for Physicians Treating Patients with Biochemical Recurrence of Prostate Cancer

**DOI:** 10.1155/2020/1929565

**Published:** 2020-04-26

**Authors:** Benjamin H. Lowentritt, Michael S. Kipper

**Affiliations:** ^1^Chesapeake Urology, Pavilion North, GBMC Campus, Tulip Park, 6535 N. Charles Street, Suite 500, Towson, MD 21204, USA; ^2^Genesis Healthcare, 3444 Kearny Villa Rd, Suite No. 201, San Diego, CA 92123, USA

## Abstract

The positron emission tomography (PET) tracer ^18^F-fluciclovine has seen increasing use to localize disease in men with biochemical recurrence of prostate cancer, i.e., elevated prostate-specific antigen (PSA) levels post-treatment. ^18^F-Fluciclovine PET/computed tomography (CT) imaging reports now play central roles in many physician-patient discussions. However, because no standardized grading system or templates yet exist for ^18^F-fluciclovine image assessment, reports vary in format, comprehensiveness, and terminology and may be challenging to fully understand. To better utilize these documents, referring physicians should be aware of six key features of ^18^F-fluciclovine PET/CT. First, ^18^F-fluciclovine is a radiolabeled synthetic amino acid targeting the amino acid transporters ASCT2 and LAT1, which are ubiquitous throughout the body, but overexpressed in prostate cancer. Second, ^18^F-fluciclovine image interpretation is predominantly visual/qualitative: radiotracer uptake in suspicious lesions is compared with uptake in bone marrow or blood pool. Location of ^18^F-fluciclovine-avid lesions relative to typical recurrence sites and findings elsewhere in the patient are considered when evaluating lesions' probability of malignancy, as is visibility on maximum intensity projection images when assessing bone lesions. Third, ^18^F-fluciclovine PET/CT detection rates increase as PSA levels rise. Fourth, detection rates may differ among centers, possibly due to equipment and reader experience. Fifth, since no diagnostic test is 100% accurate, scan data should not be used in isolation. Lastly, ^18^F-fluciclovine PET/CT findings frequently induce changes in disease management plans. In the prospective multicenter LOCATE and FALCON studies, scans altered management plans in 59% (126/213) and 64% (66/104) of patients, respectively; 78% (98/126) and 65% (43/66) of changes, respectively, involved modality switches. Referring physicians and imagers should collaborate to improve scan reports. Referrers should clearly convey critical information, including prescan PSA levels, and open clinical questions. Imagers should produce reports that read like consultations, avoid leaving open questions, and if needed, provide thoughts on next diagnostic steps.

## 1. Introduction

For physicians treating men with suspected biochemical recurrence of prostate cancer, and for their patients, detecting sites of relapse and characterizing extent of disease provide crucial information for treatment planning. Based on diagnostic performance, often confirmed histologically [1, 2], ^18^F-fluciclovine (anti-1-amino-3-^18^F-fluorocyclobutane-1-carboxylic acid (^18^F-FACBC), Axumin®, Blue Earth Diagnostics, Burlington, MA) was approved in the US in May 2016 as a tracer for “positron emission tomography (PET) imaging in men with suspected prostate cancer recurrence based on elevated blood prostate-specific antigen (PSA) levels” post-treatment [3]. Subsequently, large multicenter prospective studies in men with low median prescan PSA concentrations in the US [4] (*N* = 213; median PSA 1.0 ng/mL) and the UK [5] (*N* = 104; median PSA 0.79 ng/mL) showed high lesion detection rates (57% (122/213) and 56% (58/104)), respectively, on a per-patient basis). These studies also demonstrated that ^18^F-fluciclovine imaging changed planned patient management in the majority of cases (59% (126/213) and 64% (66/104), respectively). These findings were based on questionnaires completed by treating physicians before and after the ^18^F-fluciclovine study [4, 5]. As a result of regulatory approval and observations of diagnostic efficacy [1, 2] and clinical utility [4, 5], ^18^F-fluciclovine PET/computed tomography (CT) has seen increasing use.

With this new tool come new challenges. Among these challenges are fully understanding ^18^F-fluciclovine PET/CT reports, which play a central role in physician-patient discussions. Many urologists, for instance, have had relatively limited experience with PET, which has not been widely applied in prostate, kidney, or bladder cancers. Further, while standardized procedures exist for ^18^F-fluciclovine PET/CT image acquisition [3, 6] and image interpretation [6–8], no standardized grading system or templates have been introduced for ^18^F-fluciclovine PET/CT reporting. Thus, ^18^F-fluciclovine PET/CT reports can vary significantly in format, level of detail, and terminology. Indeed, similar to some bone scan reports, these documents may have language lacking specificity, clarity, or both [9]. Understanding ^18^F-fluciclovine PET/CT reports can be particularly challenging when they involve patients who have not undergone prostatectomy. In these men, radiation-damaged tissue, implanted radioactive seeds, inflammation, or coexisting benign prostatic hyperplasia may decrease lesion conspicuity in the prostate region, confounding image interpretation [10].

This review article addresses these challenges by sharing perspectives and practical recommendations from a urologist (BHL) and an imager (MSK) in US community practice, supplemented by insights from current literature. The first author is an experienced ^18^F-fluciclovine PET/CT referrer; the second author has extensive clinical trial and “real-world” experience acquiring, interpreting, and reporting on ^18^F-fluciclovine PET/CT images.

We first review key features of ^18^F-fluciclovine PET/CT that form the groundwork for and demonstrate the importance of, thoroughly understanding ^18^F-fluciclovine PET/CT reports. Next, we summarize published data regarding ^18^F-fluciclovine PET/CT diagnostic performance in nonprostatectomy patients, as noted above, a challenging scan setting. We conclude by offering suggestions on how referring physicians and imagers can work together to enhance understanding of ^18^F-fluciclovine PET/CT reports and to improve these documents.

## 2. Key Aspects of ^18^F-Fluciclovine PET/CT

Physicians may find it helpful to be aware of six key features of PET/CT with ^18^F-fluciclovine. First, as a radiolabeled synthetic amino acid, ^18^F-fluciclovine targets the amino acid transporters ASCT2 and LAT1 on cell surfaces [11, 12]. These transporters are overexpressed in prostate cancer and certain other malignancies and are ubiquitous throughout the body. Hence, besides uptake in lesions, there are differing degrees of physiologic uptake in healthy tissues, including the liver, bone marrow, lung, myocardium, pancreas, pituitary and salivary glands, bowel, and muscles [8, 13]. The extent and patterns of this uptake may vary according to both the tissue type and the time point post-tracer administration.

Second, ^18^F-fluciclovine image interpretation is predominantly visual/qualitative, unlike ^18^F-fluorodeoxyglucose image interpretation, which often relies more on semiquantitative standardized uptake values (SUVs). The visual/qualitative nature of ^18^F-fluciclovine image interpretation increases the need for specific patient history in study requisitions, to help increase diagnostic accuracy.

**Figure 1:**
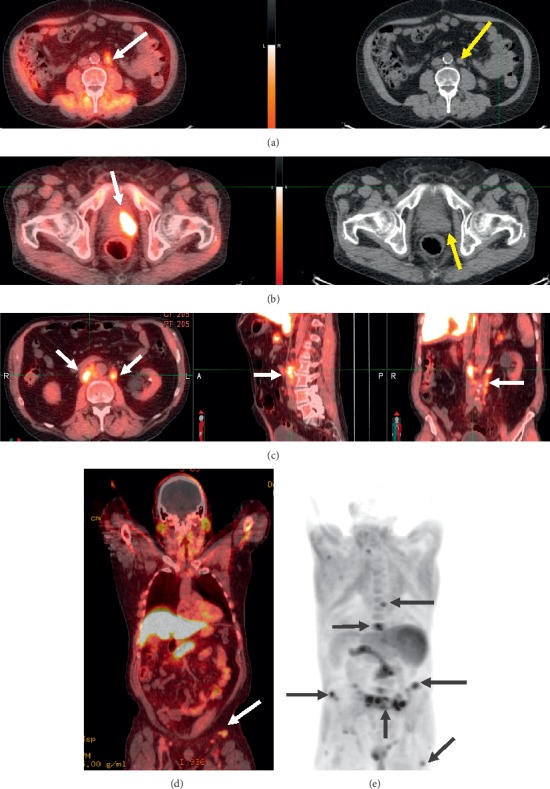
Elements recommended for determining likelihood of prostate cancer in given lesions (identified by arrows). (a) Comparison of fluciclovine uptake in nonosseous lesions to background; this is a para-aortic lymph node on fused axial image (left) and CT (right). (b) Lesion located at typical site of prostate cancer recurrence in the prostate gland in a patient given radiotherapy as initial definitive treatment (left image is axial fused and right image is corresponding CT). (c) Lesion located at typical site in lymph nodes (left image is axial fused, middle image is sagittal, and right image is coronal view). (d) Lesions located at atypical site for prostate cancer (lymph nodes). (e) Bone lesions as seen on an MIP image.

Likelihood of prostate cancer in ^18^F-fluciclovine-avid lesions is assessed based on four main elements Figure 1:

1. Comparison of radiotracer uptake in nonosseous ^18^F-fluciclovine-avid lesions with ^18^F-fluciclovine uptake in background, such as bone marrow or blood pool (Figure 1(a))
2. Location of ^18^F-fluciclovine-avid lesions relative to typical sites of prostate cancer recurrence (Figure 1(b) and 1(c))
3. Consideration of ^18^F-fluciclovine PET/CT findings elsewhere in the patient, when evaluating ^18^F-fluciclovine-avid lesions in sites atypical for recurrence (Figure 1(d))
4. Visibility on maximum intensity projection (MIP) images, when characterizing ^18^F-fluciclovine-avid bone lesions (Figure 1 (e))

For ^18^F-fluciclovine-avid lesions ≥1 cm in diameter, uptake visually equal to or greater than that in the bone marrow in L3 or in a vertebra near the lesion is a finding suggesting cancer [6]. L3 is recommended as a comparator because relative to other vertebrae, it is less likely to exhibit arthritic changes that could confound interpretation of tracer uptake. However, if uptake in L3 is not physiological, the normal vertebra nearest to the lesion should be used as a comparator.

For lesions <1 cm, ^18^F-fluciclovine visual uptake significantly greater than that in the blood pool (i.e., approaching that in bone marrow) is a finding suspicious for malignancy. Lesions <1 cm typically exhibit lower radiotracer uptake than do their larger counterparts, due to the “partial volume effect” seen in imaging in general [14]. Therefore, blood pool, which is typically a lower-uptake comparator than bone marrow, serves as the reference for smaller lesions. For lesions in the prostate bed of postprostatectomy patients, or in lymph nodes in all patients, presence in a typical site of recurrence is a principal sign suggesting malignancy. Conversely, mild, symmetric uptake in nodes where recurrence is unusual, i.e., the inguinal, distal external iliac, hilar, or axillary nodes, typically is considered to be physiologic. However, “atypical” nodal uptake occurring within the context of other clearly malignant findings is classified as being suggestive of recurrence.

An additional point regarding ^18^F-fluciclovine PET/CT scan interpretation is that prostate cancer cannot be ruled out in bone lesions seen only on CT; this situation is due to osteoblastic lesions frequently lacking enhanced ^18^F-fluciclovine uptake. When bone lesions are visualized on the CT component but not on the ^18^F-fluciclovine component of the study, the clinical situation may provide helpful information, e.g., bone lesions are uncommon when PSA is <10 ng/mL. Additionally, experienced readers may be able to identify malignant lesions based on CT appearance alone. Nonetheless, to increase diagnostic certainty, follow-up bone scan or magnetic resonance imaging (MRI) is recommended, or finally, the lesion can in some cases be biopsied. Of interest, prostate-specific membrane antigen (PSMA) radioligand tracers and ^18^F-fluorocholine also appear to exhibit a lack of enhanced uptake by some sclerotic bone lesions in patients with prostate cancer, based on comparisons with ^18^F-sodium fluoride [15–17].

**Figure 2:**
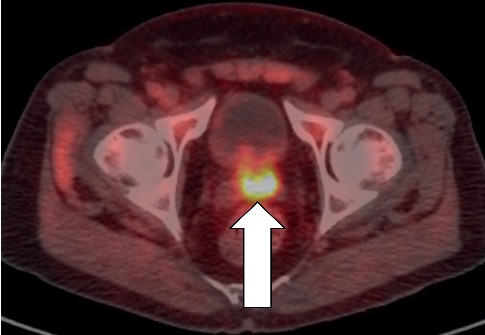
Image of a 72-year-old male who had undergone Calypso-guided intensity-modulated radiotherapy and cyberknife treatment of a right-sided iliac lymph node (identified on a prior ^18^F-fluciclovine study). He was referred for ^18^F-fluciclovine imaging with PSA rising to 15.1 ng/mL. This axial, fused PET/CT image shows intense ^18^F-fluciclovine uptake in the left side of the prostate gland (white arrow), consistent with prostate cancer recurrence.

**Figure 3:**
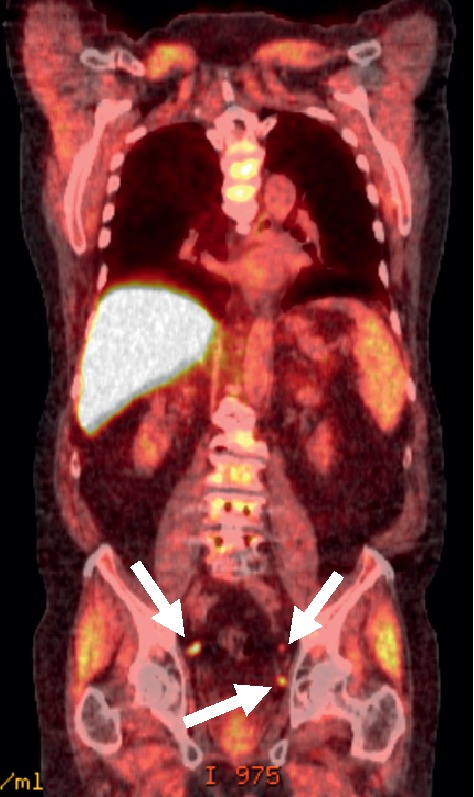
Image of an 81-year-old male who had received proton beam radiation therapy. He was referred for ^18^F-fluciclovine imaging with PSA rising to 3.3 ng/mL. This coronal, fused PET/CT view shows 3 prostate cancer metastases (white arrows). Note the node superiorly on the right, with very subtle ^18^F-fluciclovine uptake; this node is ∼3 mm.

**Figure 4:**
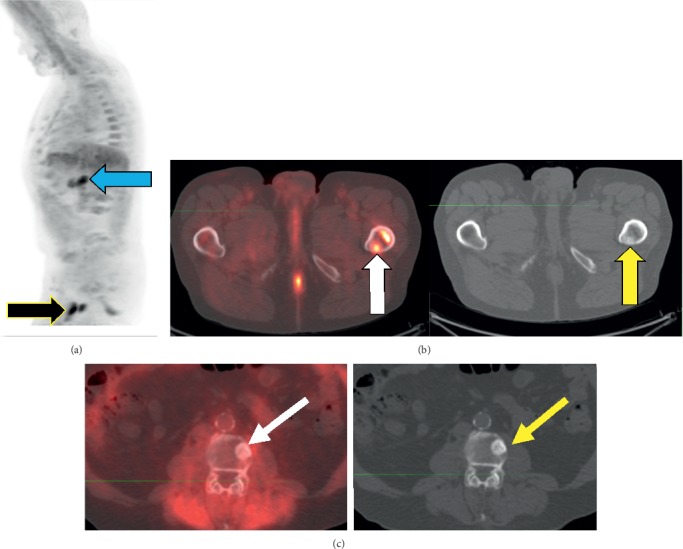
Images of a 79-year-old male who had undergone radical prostatectomy, salvage radiation, and proton beam therapy for oligometastases to L3 and multiple lymph nodes. He was referred for ^18^F-fluciclovine imaging with PSA rising to 2.03 ng/mL. (a) MIP view, sagittal plane. The black arrow points to enhanced uptake, consistent with metastasis in the proximal left femur. The blue arrow points to the pancreas (normal physiologic uptake). (b) Axial, fused PET/CT image (white arrow) and corresponding CT view (yellow arrow) show a metastasis in the proximal left femur. (c) Of interest, this PET/CT view and the corresponding CT view show a previously-irradiated metastasis in L3; note the absence of ^18^F-fluciclovine uptake (white arrow) on PET and presence of sclerosis on CT (yellow arrow).

**Figure 5:**
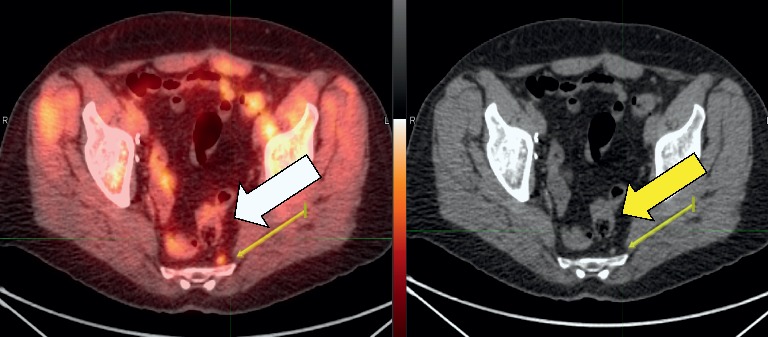
Images of a 68-year-old male with T3N0M0 prostate cancer. He had undergone radical prostatectomy, bilateral lymph node dissection, and salvage radiation therapy. He was referred for ^18^F-fluciclovine imaging with PSA rising to 0.7 ng/mL. The axial, fused PET/CT image on the left identifies a focus of enhanced ^18^F-fluciclovine uptake (white arrow) in the presacral region. The corresponding CT image on the right identifies a 6-mm lymph node metastasis (yellow arrow).

**Figure 6:**
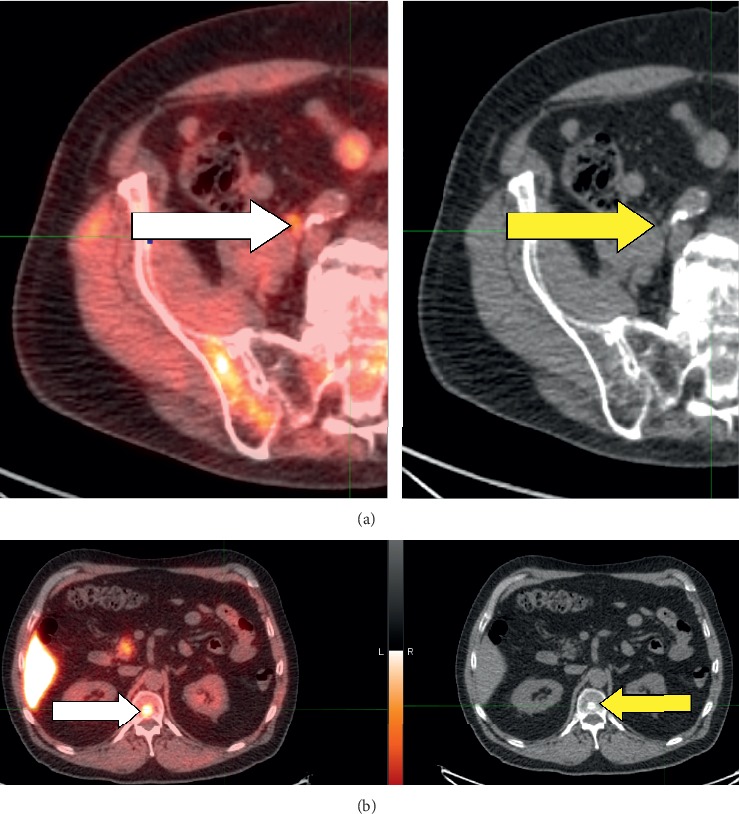
Images of an 80-year-old male who had received robotic-assisted laparoscopic prostatectomy and salvage radiation therapy. He was referred for ^18^F-fluciclovine imaging with PSA rising to 1.1 ng/mL. Top row: The axial, fused PET/CT image on the left identifies a focus of mildly enhanced ^18^F-fluciclovine uptake (white arrow) at the level of the common iliac vessels. The corresponding CT image on the right identifies 2 small, 5-mm lymph nodes (yellow arrow). Bottom row: note the ^18^F-fluciclovine-avid lesion in the body of T12 (white arrow, left-hand image), with the corresponding CT view on the right demonstrating the lesion at the identical location (yellow arrow).

**Figure 7:**
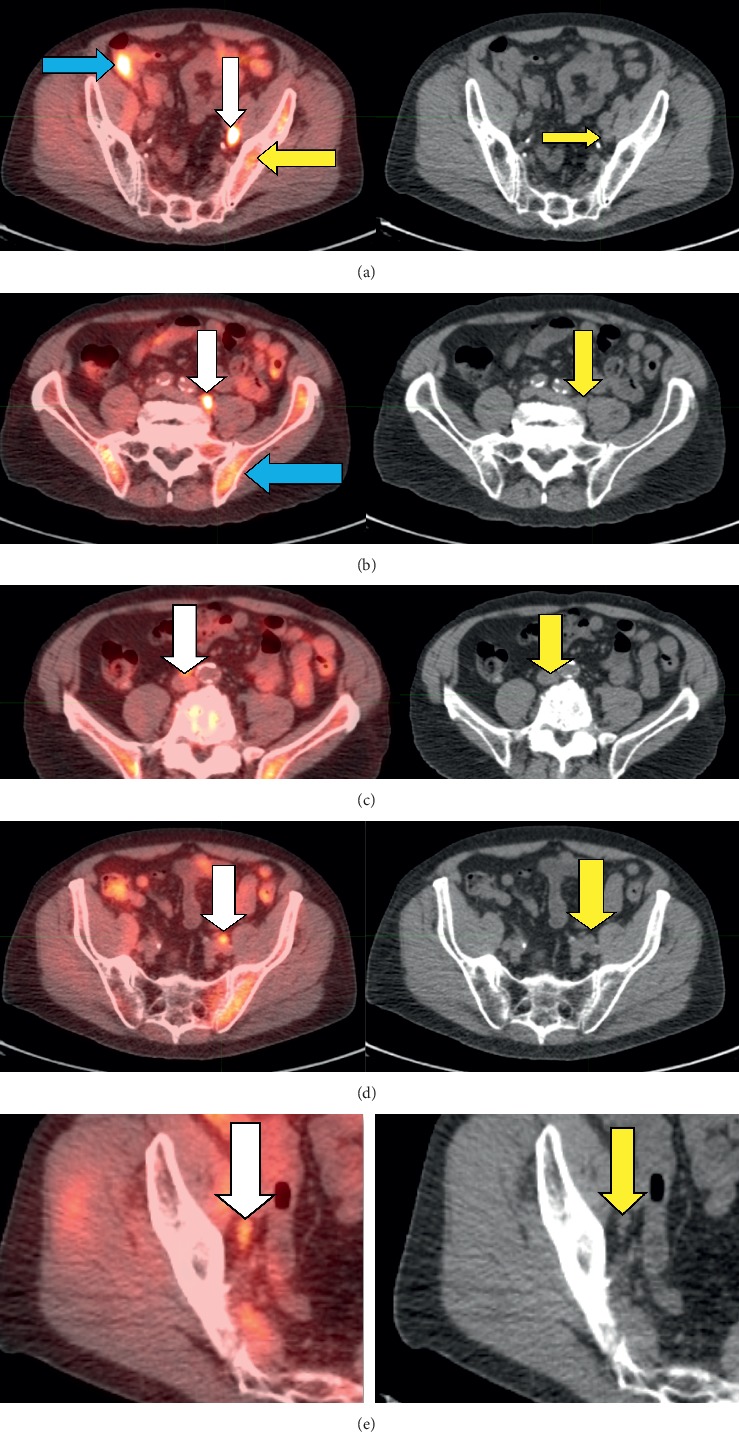
Images of a 68-year-old male who had undergone radiation therapy, followed by short-term androgen deprivation therapy. He was referred for ^18^F-fluciclovine imaging, on no treatment, when his PSA rose to 10.5 ng/mL. Five rows of images from his study are shown, to demonstrate varying intensities of uptake. SUVs, a semiquantitative measure of uptake intensity, are given, with higher values denoting greater intensity; compare to marrow uptake at the same level (yellow arrows in PET images). First (top) row: the first, left-hand image identifies a 1.4-cm left obturator node (SUV: 7.6) (white arrow). The blue arrow points to physiologic bowel uptake. The yellow arrow points to physiologic uptake in the marrow of the left iliac bone. The image on the right is the corresponding CT view; the yellow arrow points to the left obturator node. Second row down: left-hand image shows metastasis at the level of the common iliac nodes (SUV: 5.8). The blue arrow points to typical, heterogeneous bone marrow uptake (normal). The corresponding CT image on the right shows the node adjacent to the psoas muscle. Third row down: the left-hand image identifies a 1-cm aortocaval metastasis (SUV: 3.2). The yellow arrow on the corresponding CT view points to a lymph node at this site. Fourth row down: the left-hand image identifies a 1-cm left external iliac node (SUV: 3.5). The yellow arrow on the right points to the node on the CT view. Fifth (bottom) row: the left-hand image shows a 6-mm right obturator node (white arrow). Although the SUV is only 2.7, the node still clearly shows enhanced uptake compared to the adjacent iliac marrow. CT image on the right identifies the node (yellow arrow).

**Figure 8:**
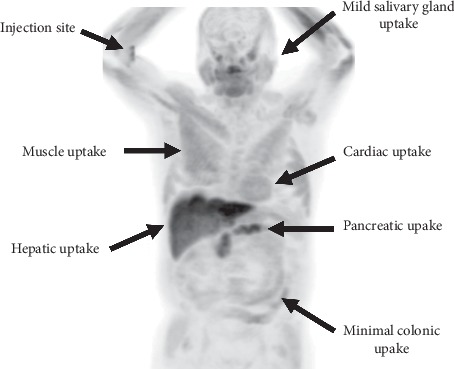
Physiologic uptake of ^18^F-fluciclovine. This is an MIP view from a negative study. Additional sites of physiologic/benign ^18^F-fluciclovine uptake may occur in the pituitary gland, the kidneys, ureters, bladder, ganglia, and adrenals. Note the absence of bladder activity in this patient.

^18^F-Fluciclovine PET/CT image interpretation is described in detail elsewhere [7, 8]. Additionally, interpretation can be studied in an online training module of the Society for Nuclear Medicine and Molecular Imaging. Directed towards nuclear medicine physicians and radiologists, but also helpful for frequent ^18^F-fluciclovine PET/CT referrers, the module is accessible at snmmilearningcenter.org/Activity/4521746/Detail.aspx (last accessed 19 April 2020). Representative ^18^F-fluciclovine PET/CT images are shown in Figures 2–8.

A third “need-to-know” regarding ^18^F-fluciclovine PET/CT is that detection rates increase along with rising PSA levels [2, 4] (Table 1). Hence, providing scan readers with the PSA data of patients undergoing ^18^F-fluciclovine imaging is particularly important.

Fourth, ^18^F-fluciclovine PET/CT disease detection rates may differ among centers, particularly at PSA levels ≤1.0 ng/mL [18] (Supplementary Table S1). For example, in LOCATE, the previously mentioned 213-patient, 15-center US study [4], center-to-center differences in the median PSA levels of the respective cohorts, and in frequency of prostatectomy, i.e., differences in the case mix, likely were important contributors to variability in detection rates. Other possible contributory factors that should always be considered include the following: the make/model, age, and features of the PET scanner, and readers' training and experience in PET scan interpretation.

Fifth, since no diagnostic test is 100% accurate, a negative ^18^F-fluciclovine PET/CT scan does not rule out, and a positive scan does not confirm prostate cancer. ^18^F-Fluciclovine scan data therefore should not be used in isolation from the clinical context of the patient, the clinical judgment and experience of the treating physician, or the results of other imaging or of histopathological testing.

Lastly, as noted above, ^18^F-fluciclovine PET/CT findings have been shown to lead to frequent, and often important, changes in disease management plans [4, 5, 19, 20]. In LOCATE, these alterations were classified as major, i.e., involving additions, subtractions, or both of a treatment modality or modalities, in 98/126 cases with changes (78%; 46% of evaluable patients). The changes were classified as minor, i.e., involving adjustments in the regimen of a modality, in 28/126 cases (22%). Management plan changes were attributable to positive scans in 88/126 patients (70%) and to negative scans in 38/126 (30%).

The LOCATE cohort comprised men with increasing PSA levels following curative-intent primary treatment with one or more of radical prostatectomy, radiotherapy, or other systemic or local interventions. Prescan PSA ranged from 0.2 to 93.5 ng/mL. Notably, negative or equivocal conventional imaging (bone scan and/or abdominal/pelvic CT or MRI) in the 60 days preceding trial entry was a requirement for inclusion. One of the authors (MSK) was an investigator in LOCATE.

FALCON, a 6-center UK study [5], including 104 evaluable patients with biochemical recurrence of prostate cancer, had findings that aligned with those of LOCATE [4]. Among men with scan-related changes in management plans (64%, 66/104), those alterations were classified as major, i.e., entailing switches in treatment modality, in just under 2/3 of cases (65%, 43/66), and as “other,” i.e., entailing adjustments of a treatment modality's regimen, in the remaining cases (35%, 23/66). Management plan revisions were attributable to a positive scan in 80% (53/66) of cases and to a negative scan in 20% (13/66). Of interest, a secondary analysis of FALCON suggested that ^18^F-fluciclovine imaging-guided salvage therapy was associated with a higher short-term PSA response rate than was salvage therapy not guided by such imaging (88% (15/17) vs. 72% (28/39)).

The study sample in FALCON ranged in prescan PSA level from 0.04 to 28.0 ng/mL and, like that in LOCATE, had undergone curative-intent primary treatment with one or more of radical prostatectomy, radiotherapy, or other systemic or local interventions. Unlike LOCATE, FALCON had no entry requirement of negative or equivocal conventional imaging.

## 3. ^18^F-Fluciclovine PET/CT in Patients with Intact Prostates

Limited published information is available on ^18^F-fluciclovine PET/CT in men with intact prostates and biochemical failure. A small, single-center prospective study [10] including only such patients (*N* = 24) found high sensitivity (100%) but low specificity (11%) for disease detection in the prostate (*n* = 22 evaluable). Both sensitivity (88%) and specificity (90%) were high for detection outside the prostate (*n* = 18 evaluable).

In another prospective study [1] with 74% (69/93) nonprostatectomy patients, ^18^F-fluciclovine PET/CT sensitivity and specificity, respectively, were 90% and 40% in the prostate/prostate bed (*n* = 91) and 55% and 97% for extraprostatic disease (*n* = 70).

A third prospective study, reported in 2019 [21, 22], involved 21 consecutive nonprostatectomy patients with rising PSA (median (minimum − maximum) 4.5 (1.0–26.7) ng/mL) after definitive treatment with radiotherapy (43%), cryotherapy (14%), or both (43%). In this small, single-center trial, the detection rate for disease in the prostate for experimental ^18^F-fluciclovine PET/CT/ultrasound fusion biopsy was 48% (10/21 patients). Conventional transrectal prostate ultrasound template biopsy failed to detect prostatic recurrence in 5 of these 10 patients, but found such disease in 1 case missed by the ^18^F-fluciclovine PET/CT-aided procedure. The ^18^F-fluciclovine scan also had a 38% (8/21) detection rate for extraprostatic disease (nodal in 7 cases, osseous in 1).

LOCATE included 49 patients with intact prostates, 23% of the evaluable cohort; 164 patients (77%) had undergone prostatectomy [23]. Overall scan positivity rates were higher in patients with an intact prostate than in their counterparts who underwent prostatectomy: 84% vs. 49%; the difference appeared to be almost completely attributable to positivity rates in the prostate region.

Referring and interpreting physicians should know that according to standard ^18^F-fluciclovine PET/CT image interpretation methodology [6, 7], the principal criterion to diagnose recurrence in the prostate bed of men with intact prostates is diffuse, focal, or multifocal ^18^F-fluciclovine uptake visually equal to or greater than bone marrow uptake. When lesions are <1 cm, the principal criterion to diagnose recurrence is ^18^F-fluciclovine uptake visually significantly greater than uptake in the blood pool (i.e., approaching that in bone marrow).

## 4. Improving ^18^F-Fluciclovine PET/CT Reports and Referrer-Reader Communication

Both referring physicians and imagers can help improve the quality of ^18^F-fluciclovine PET/CT reports. Insofar as possible, referring physicians should seek imagers who have had specific training in ^18^F-fluciclovine PET/CT acquisition and interpretation [7]. Further, referring physicians should furnish imagers with clinical/laboratory data that will help optimize the study report. Most importantly, referrers should provide readers with the clinical question(s) to be answered by the scans. Appendix provides an example of a referral request, which addresses the items of most value to the image reader. Inclusion of the most recent note(s) from the patient record can also be extremely helpful.

It is useful to bear in mind key elements of a good imaging report. The report should read like a consultation, not a “summary of hotspots.” The document should avoid leaving open questions and, in those cases where additional investigation may be warranted, should provide thoughts on next diagnostic steps. Additionally, reports can be enhanced by including one or more color images of key findings, as well as a description of these findings' location. An example of such a description would be, “There is a focus of enhanced ^18^F-fluciclovine uptake in the right external iliac region, with a 1.4 cm lymph node, identified on the correlative CT portion of the study (axial image 236).” For referring physicians wishing access to all scan data, Health Insurance Portability and Accountability Act-compliant connectivity tunnels can be established for image/data transfer; for those wishing selected data, studies can be placed on disk, typically including an embedded program for reading.

## 5. Role of ^18^F-Fluciclovine versus Other “Advanced” PET Tracers

In the rapidly evolving field of imaging patients with biochemical recurrence of prostate cancer [24, 25], ^18^F-fluciclovine is, along with choline and PSMA ligand radiopharmaceuticals, an “advanced” or “next-generation” radiotracer that has gained broad clinical usage. Literature comparing the diagnostic performance or detection rates of ^18^F-fluciclovine against those of choline tracers labeled with ^18^F or ^11^C, or PSMA ligand tracers, is limited. An Italian group found superior detection rates for ^18^F-fluciclovine PET/CT versus ^11^C-choline PET/CT in a prospective study involving 100 patients with biochemical recurrence after radical prostatectomy [26–29]. In a report [30] on 89 of these patients, these investigators noted statistically significant differences favoring ^18^F-fluciclovine in true-positive, true-negative, false-positive, and false-negative findings. They also noted that across PSA categories, ^18^F-fluciclovine tended to be associated with more frequent true-positive findings than was ^11^C-choline; the difference was statistically significant in patients with PSA <1 ng/mL (subgroup size not reported). These observations, as well as practical advantages in radiopharmaceutical handling and ease of scan interpretation, led the authors to describe ^18^F-fluciclovine as “an alternative tracer superior to ^11^C-choline” in the setting of biochemical recurrence following radical prostatectomy [30]. In the study, PET/CT with each tracer took place within 1 week, and the reference standard was clinical follow-up at 1 year, including any available result(s) of additional imaging, PSA measurement, and pathological examination.

Two published prospective paired-scan comparisons [31, 32] of ^18^F-fluciclovine PET/CT versus ^68^Ga-PSMA-11 PET/CT showed mixed results. A University of California Los Angeles (UCLA) study involving 50 men with biochemical recurrence after radical prostatectomy and prescan PSA <2.0 ng/mL noted significantly lower detection rates with ^18^F-fluciclovine overall (patient-level) and in the pelvic node and extrapelvic regions. In this study, the scans were performed within ≤15 days, and the reference standard was a composite of histopathology or additional imaging or PSA changes during a median follow-up of 8 months. Some limitations of this study were noted [33]: ^18^F-fluciclovine imaging tended to have been started at an earlier-than-recommended timepoint after tracer administration, and was performed elsewhere than at UCLA in 24% of patients (12/50), and ^18^F-fluciclovine images were interpreted by less experienced readers than were the ^68^Ga-PSMA-11 scans.

By contrast, an Austrian study [32] (N = 58) found statistically similar overall detection rates for the two tracers (79% vs. 83%, *p* = 0.64) and a significantly higher detection rate for local recurrence with ^18^F-fluciclovine (38% vs. 28%, *p* = 0.03). The authors attributed the latter finding to ^68^Ga-PSMA-11 accumulation in the urinary bladder, which would be expected to confound prostate/bed lesion detection. In this study, the scans took place a mean 9.4 days apart. Mean (minimum–maximum ) prescan PSA was 14.9 (0.2–230.4) ng/mL, and primary treatment comprised radical prostatectomy in 72% of patients and radiotherapy in 28%.

Given the limited comparative literature, the respective roles going forward of ^18^F-fluciclovine and of the choline and PSMA ligand tracers will depend on commercial availability, cost, and reimbursement. PSA level at the time of the scan also may factor into decision-making, as there is a perception that PSMA radioligands may perform better than do the other tracers at very low PSA levels. However, as noted above, evidence for that perception is limited, and indeed, the impact on outcome, if any, of disease detection at very low versus low PSA levels remains unknown.

## 6. Conclusions

As shown by LOCATE and FALCON, ^18^F-fluciclovine PET/CT findings can profoundly affect prostate cancer management. Therefore, it is of paramount importance for treating physicians to fully understand ^18^F-fluciclovine PET/CT reports, and for referrers and readers to work together to optimize image interpretation and reporting. Referring physicians should educate themselves on the basics of ^18^F-fluciclovine PET/CT image interpretation and on the value and limitations of ^18^F-fluciclovine PET/CT disease detection. They should seek out imagers trained in ^18^F-fluciclovine image acquisition and interpretation, and should clearly convey to imagers the critical information needed for individual cases. Finally, both referrers and readers should remember that there is no substitute for direct communication!

## Figures and Tables

**Table 1:** Patient-level prostate cancer detection rate by PSA category, LOCATE study (*N* = 213 evaluable patients) [19].

PSA concentration category	Overall (patient-level) prostate cancer detection rate
0–0.5 ng/mL	31% (25/81)
>0.5–1.0 ng/mL	50% (13/26)
>1.0–2.0 ng/mL	66% (19/29)
>2.0–5.0 ng/mL	77% (27/35)
>5.0–10.0 ng/mL	87% (20/23)
>10.0 ng/mL	95% (18/19)

PSA, prostate-specific antigen.

## Appendix

Example of an ^18^F-fluciclovine PET/CT referral request is given, which addresses the items of most value to the image reader.

## Supplementary Materials

Supplementary Materials Supplementary Table 1: range of patient-level cancer detection rates by ^18^F-fluciclovine PET/CT for 5 centers with the most patients in the LOCATE study. Appendix—Suggested 18F-fluciclovine PET/CT requisition form.
