# Supplementary material for: Understanding and Improving 18F-Fluciclovine PET/CT Reports: A Guide for Physicians Treating Patients with Biochemical Recurrence of Prostate Cancer
Source: Prostate Cancer. 2020 Apr 26;2020:1929565. doi: 10.1155/2020/1929565 (PMC7199579; doi:10.1155/2020/1929565)
Supplement: Supplementary Materials — Supplementary Table 1: range of patient-level cancer detection rates by 18F-fluciclovine PET/CT for 5 centers with the most patients in the LOCATE study. Appendix—Suggested 18F-fluciclovine PET/CT requisition form. [file 1929565.f1.pdf]

Supplementary Tables

Supplementary Table S1. Range of patient-level prostate cancer detection rates for the 5 centers contributing >20 patients per center to the LOCATE study[14] (n = 137/213 evaluable patients)

**Group**	**Range of patient-level detection rates, % (n)**
All patients (n = 21–33 per center)	55% (18/33)–77% (24/31)
Patients with PSA ≤1 ng/mL (n = 10–16 per center)	20% (2/10)–71% (10/14)

PSA, prostate-specific antigen

Appendix 1

^{18}F-Fluciclovine (Axumin) Imaging Request

Patient Name: ____ DOB: _________________
Referral Physician: _____________________________________ MRN: ________________

Reason(s) for Study Request:

- **Biochemical Recurrence (BCR): ____**
- **Other: ____**

Initial Therapy (please include date)

- **Prostatectomy ___________________**
- **Radiation _______________________**
- **Other ____**

Two most recent PSA measurements (please include dates) __________________________________

Please provide the following if available: reports of MRIs, CT studies, bone scans, and ultrasounds, pathology reports, and most recent physician office visit

Please instruct patient to be NPO (except water and medications) for 4 hours prior to study, and to refrain from strenuous exercise for 24 hours prior to study; please provide patient with medication if claustrophobic

Thank you very much for your kind referral

Please check those items below which would be of help to your office

____ Would your doctor appreciate a call with the results? (phone number) _______________
____ Are there additional physicians you would like this report to go to? _________________
____
____ Is there a contact person at your office for questions? _____________________________

ABC Imaging
3456 Washington Street, Suite 201
San Diego, CA 92111
Phone: ( ) _ _ _ - _ _ _ _ Ext. _____
Fax: ( ) _ _ _ - _ _ _ _
E-mail:
Chief Technologist: __________________________ Phone: ( ) _ _ _ - _ _ _ _ Ext. ________
